# Supplementary material for: Do metrics of sexual selection conform to Bateman's principles in a wind-pollinated plant?
Source: Proc Biol Sci. 2019 Jun 19;286(1905):20190532. doi: 10.1098/rspb.2019.0532 (PMC6599987; doi:10.1098/rspb.2019.0532)
Supplement: Supplementary Methods S1 [file rspb20190532supp1.pdf]

Supplementary methods S1 : decomposition of variance in male reproductive success in plants

Unlike female reproductive success, male reproductive success is not usually measured directly, but indirectly by counting the number of female mates for each male (male mating success), and then by determining how many offspring a male gains from each of his mates. Thus, previous work [34,35] decomposed male mating success (RS_m) as a product of three components.

$$RS_m = MS_m \times f \times p \qquad \text{(Eq1)}$$

where MS_m is male mating success (the number of females bearing at least one offspring of a particular male), f is the average fecundity (seed production) of a female pollinated by a particular male, and p is the average paternity share of a male on each of his female mates (a weighted average, each female weighted by her fecundity). An approximation of the male opportunity for selection (variance in relative male RS_m) is then given by :

$$V(RS_m^*) \approx V(MS_m^*) + V(f^*) + V(p^*) + 2COV^* \qquad \text{(Eq2)}$$

where the star superscript denotes relativized values (divided by the grand mean) and the COV^* term represents the sum of the three pairwise covariances between the relativized components [34,35]. The approximation will work better if the variances are not too high compared to their respective means, and may be specially imprecise when there are males with low or null MS_m. As paternity share and fecundity of female partners cannot be calculated when there are no female mates, only males with MS > 0 are considered to compute their variances and covariances.

In the context of our plant experiment, the peculiar spatial arrangement of plants makes it useful to distinguish two sources of reproductive success for each male. This is because each male has privileged access to one particular female planted in the same pot ('intrapair' female, distance zero), while all 99 other females are more or less distant, and each paired with another male. This situation is in some respects similar to socially monogamous birds with a mix of within- and extra-pair paternities for each male. Because these two components of male fitness are not necessarily strongly associated, it is useful to decompose male RS_m into :

$$RS_m = RS_{m,intra} + RS_{m,extra} \qquad \text{(Eq3)}$$

which gives

$$RS_m^* = \alpha RS_{m,intra}^* + (1-\alpha)RS_{m,extra}^* \quad \text{(Eq4)}$$

where α is the overall fraction of extra-pair paternity in the data.

This immediately provides a simple decomposition of total male opportunity for selection into an intrapair variance (males that are more or less able to monopolize paternities from their local mates), an extrapair variance (males more or less able to sire seed on distant females) and a covariance between the two (depending on whether the same males tend to obtain many offspring at both scales) :

$$V(RS_m^*) = \alpha^2 V(RS_{m,intra}^*) + (1-\alpha)^2 V(RS_{m,extra}^*) + 2\alpha(1-\alpha)COV(RS_{m,intra}^*, RS_{m,extra}^*).$$

(Eq5)

Each of the variance terms (intrapair and extrapair) can in turn be decomposed using Eq(2). This is quite simple at the extrapair level, in which mating success can take several of different values (in our design, from 0 to 99). The extrapair component of the male opportunity for selection can therefore be decomposed usefully into a variance in extrapair mating success (mate acquisition), a variance in the average seed production of extrapair mates (males that pollinate more fecund females have a fitness advantage, whether by chance or not), a variance in the extrapair paternity share, and covariances among these three terms. However, the same approximation at the intrapair scale is made imprecise by the binary distribution of intrapair male mating success (1 if the local female shares some offspring with the male, 0 if not). In that case, a better approximation than (Eq 2) is obtained by conditioning on male mating success. Noting q the proportion of males that share some offspring with their local female (MS_{intra}>0), we have :

$$V(RS_{m,intra}^*) = \frac{1-q}{q} + \frac{1}{q}V(\widetilde{RS}_{m,intra}^*) \quad \text{(Eq.6)}$$

where the first variance component (1-q)/q corresponds to the failure/success of having managed to sire offspring of the local mate or not, and the second component is the remaining variance in offspring number, computed only on successful males (this condition being denoted by a tilde in $\widetilde{RS}$. This second component can in turn be decomposed into a variance in the

fecundity of the local female, a variance in the proportion of offspring sired (when >0), and a covariance between the two

$$V(\widetilde{RS}^*_{m,intra}) \approx V(\tilde{f}^*_{m,intra}) + V(\tilde{p}^*_{m,intra}) + 2COV(\tilde{f}^*_{m,intra}, \tilde{p}^*_{m,intra}) \qquad \text{(Eq. 7).}$$

Note that in the case of intrapair components, all the succesful males considered to compute Eq. 7 have the same MS = 1, so the variance in MS is null, and the covariances between MS and paternity share, and between MS and mate fecundity, are undefined: only one of the three covariance term remains, between paternity share and mate fecundity.

In summary, we combine Eq 2 to 7 to get the complete decomposition of male opportunity for selection into 11 terms:

$$\begin{aligned} V(RS_m) \approx\ & \alpha^2 \left(\frac{1-q}{q} + \frac{1}{q}\left(V(\tilde{f}^*_{m,intra}) + V(\tilde{p}^*_{m,intra}) + 2COV(\tilde{f}^*_{m,intra}, \tilde{p}^*_{m,intra}) \right) \right) \\ & + (1-\alpha)^2 \left(V(MS^*_{m,extra}) + V(f^*_{extra}) + V(p^*_{extra}) + 2COV(MS^*_{m,extra}, f^*_{extra}) \right. \\ & \left. + 2COV(MS^*_{m,extra}, p^*_{extra}) + 2COV(f^*_{extra}, p^*_{extra}) \right) \\ & + 2\alpha(1-\alpha)COV(RS^*_{m,intra}, RS^*_{m,extra}). \qquad \text{(Eq8)} \end{aligned}$$

Supplementary methods S2: simulation model of pollen dispersal abilities at different spatial scales and its impact on the effect of plant density on sexual selection

We investigated the effect of plant density on the intensity of sexual selection by modelling pollen dispersal from male pollen donors to female recipients. Pollen dispersal from each male donor was simulated using a negative exponential function (similar to empirical kernels, e.g. [31,32]), with parameter λ describing the rate of decay of pollen dispersal. To calculate the reproductive success of each simulated male, we first computed the amount of pollen received by each female recipient from each male donor, then calculated the proportion of seeds sired on each female by a given male by dividing the amount of pollen that it deposited on each female by the total amount received by this female. We assumed that each female produced the same number of seeds (specifically, 1,000), and counted the number of seeds sired by each male. To estimate the mating success of each male, we simulated a sampling of eight seeds within the seed pool of each female (as was performed in our experiment). In this seed sampling step, the probability of sampling seeds from each pollen donor was weighted by its relative contribution to the pollen cloud around each female. For each male, we then calculated the effective mating success as the number of female mates on which at least one seed was counted.

We first simulated pollen dispersal with the exact spatial arrangement of our common gardens (i.e., 100 male-female pairs in a regular 10x10 plant grid at low- and high-density), we modelled pollen dispersal. Second, we simulated pollen dispersal in grid at low- and high-density, where 100 males and 100 females were randomly assigned to a position. To evaluate the effect of plant density on sexual selection, we modelled three scenarios: (1) males varied in their ability to disperse pollen, with an average pollen dispersal distance of 0.5 m, i.e., shorter than the typical inter-sexual distance at high density; (2) males varied in their ability to disperse pollen, with an average pollen dispersal distance of 10 m, i.e., further than the typical inter-sexual distance at low density; and (3) all males displayed the same pollen dispersal kernel (i.e., differences in the intensity of sexual selection as a function of density would simply result from the spatial arrangement of plants), with a pollen dispersal distance of 5.25 m. To introduce variance in male dispersal abilities, we randomly drew a dispersal distance for each male on a uniform distribution 90% on either side of the mean dispersal distance. In the simulation case where we assumed no variance between males, we used an intermediate pollen dispersal distance because: (i) applying a very long and fixed distance of pollen dispersal resulted in very low variation between males in their abilities to access mates and to reproduce; and (ii) applying a very short

and fixed distance of pollen dispersal resulted in the ability of males to mate only with their local female. For each scenario, we calculated Bateman metrics (I, I_S and β_{SS}). Simulation results are reported as means and standard deviations for these parameters over 100 simulated replicates.
