# Supplementary figures and images for: Do metrics of sexual selection conform to Bateman's principles in a wind-pollinated plant?
Source: Proc Biol Sci. 2019 Jun 19;286(1905):20190532. doi: 10.1098/rspb.2019.0532 (PMC6599987; doi:10.1098/rspb.2019.0532)

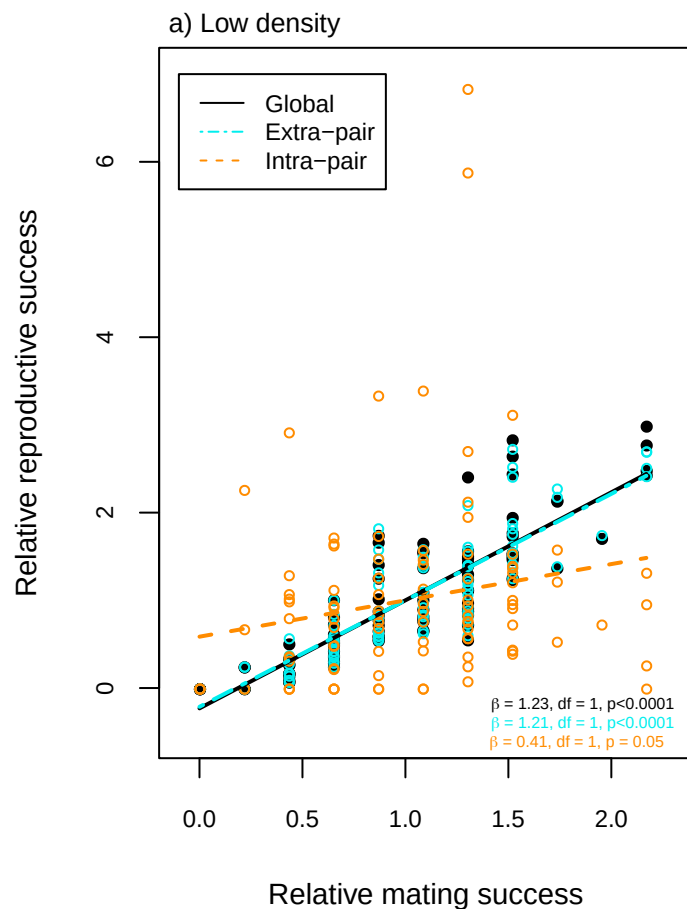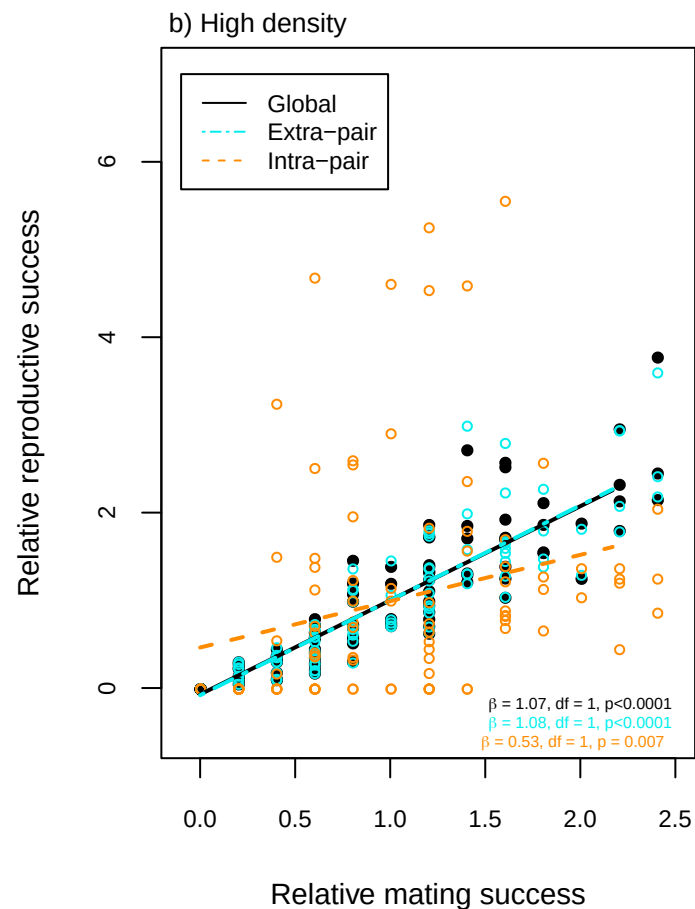

Figure S1

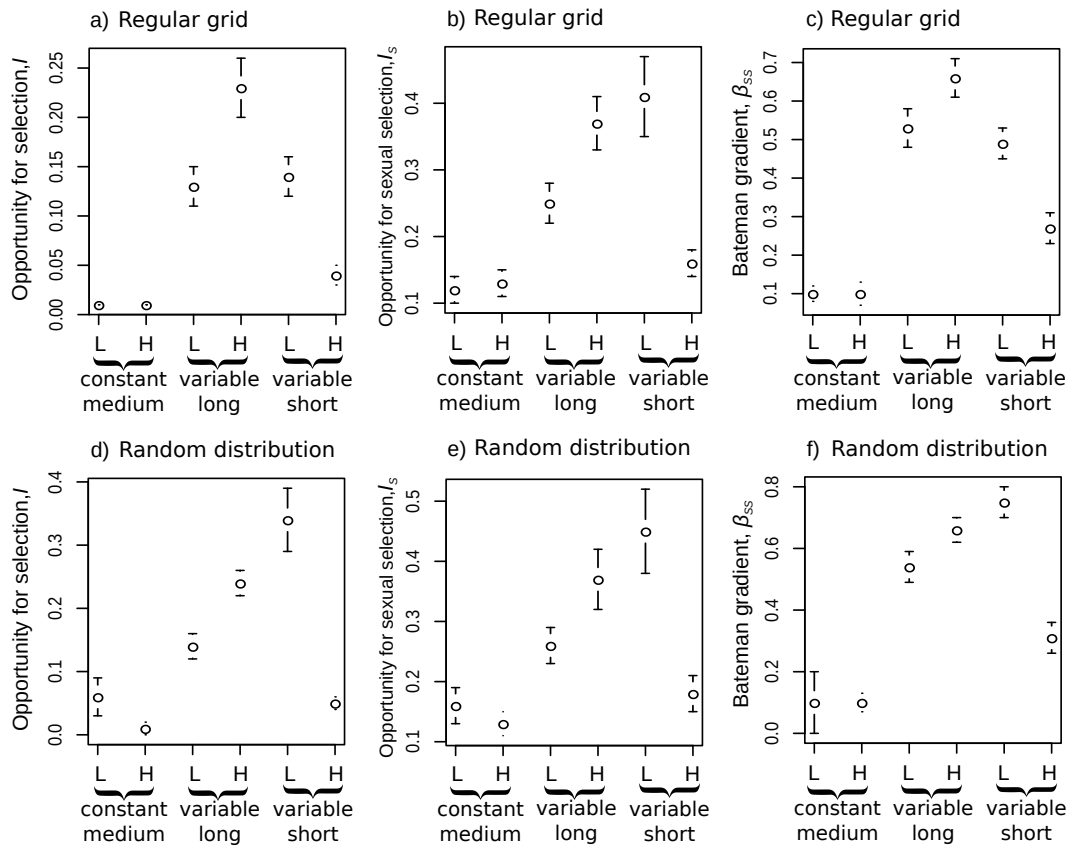

Figure S2

Supplement: Supplementary Figures S1 and S2 [file rspb20190532supp2.pdf]
